# Clinical effects of bacteremia in sepsis patients with community-acquired pneumonia

**DOI:** 10.1186/s12879-023-08887-5

**Published:** 2023-12-19

**Authors:** Tae Wan Kim, Se-Uk Lee, Boram Park, Kyeongman Jeon, Sunghoon Park, Gee Young Suh, Dong Kyu Oh, Soo Yeon Lee, Mi Hyeon Park, Haein Lee, Chae-man Lim, Ryoung-Eun Ko, Korean Sepsis Alliance (KSA) investigators, Sang-Bum Hong, Suk-Kyung Hong, Yeon Joo Lee, Young-Jae Cho, Sung Yoon Lim, Jeongwon Heo, Jae-myeong Lee, Kyung Chan Kim, Youjin Chang, Sang-Min Lee, Woo Hyun Cho, Sang Hyun Kwak, Heung Bum Lee, Jong-Joon Ahn, Gil Myeong Seong, Song-I Lee, Tai Sun Park, Su Hwan Lee, Eun Young Choi, Jae Young Moon

**Affiliations:** 1 Department of Critical Care Medicine, Samsung Medical Center, Sungkyunkwan University School of Medicine, 81 Irwon-Ro, Gangnam-Gu, Seoul, 06351 South Korea; 2 Department of Emergency Medicine, Samsung Medical Center, Sungkyunkwan University School of Medicine, Seoul, South Korea; 3 Biomedical Statistics Center, Research Institute for Future Medicine, Samsung Medical Center, Seoul, South Korea; 4 Department of Medicine, Division of Pulmonary and Critical Care Medicine, Samsung Medical Center, Sungkyunkwan University School of Medicine, Seoul, South Korea; 5 Department of Pulmonary, Allergy and Critical Care Medicine, Hallym University Sacred Heart Hospital, Anyang, South Korea; 6 Department of Pulmonary and Critical Care Medicine, Asan Medical Center, University of Ulsan College of Medicine, Seoul, South Korea; 7 Korean Society of Critical Care Medicine, Seoul, South Korea

**Keywords:** Bacteremia, Pneumonia, Community-acquired infection, Blood culture, Multicenter study

## Abstract

**Background:**

Data regarding the clinical effects of bacteremia on severe community-acquired pneumonia (CAP) are limited. Thus, we investigated clinical characteristics and outcomes of severe CAP patients with bacteremia compared with those of subjects without bacteremia. In addition, we evaluated clinical factors associated with bacteremia at the time of sepsis awareness.

**Methods:**

We enrolled sepsis patients diagnosed with CAP at emergency departments (EDs) from an ongoing nationwide multicenter observational registry, the Korean Sepsis Alliance, between September 2019 and December 2020. For evaluation of clinical factors associated with bacteremia, we divided eligible patients into bacteremia and non-bacteremia groups, and logistic regression analysis was performed using the clinical characteristics at the time of sepsis awareness.

**Result:**

During the study period, 1,510 (47.9%) sepsis patients were caused by CAP, and bacteremia was identified in 212 (14.0%) patients. Septic shock occurred more frequently in the bacteremia group than in the non-bacteremia group (27.4% vs. 14.8%; *p* < 0.001). In multivariable analysis, hematologic malignancies and septic shock were associated with an increased risk of bacteremia. However, chronic lung disease was associated with a decreased risk of bacteremia. Hospital mortality was significantly higher in the bacteremia group than in the non-bacteremia group (27.3% vs. 40.6%, *p* < 0.001). The most prevalent pathogen in blood culture was *Klebsiella pneumoniae* followed by *Escherichia coli* in gram-negative pathogens.

**Conclusion:**

The incidence of bacteremia in severe CAP was low at 14.0%, but the occurrence of bacteremia was associated with increased hospital mortality. In severe CAP, hematologic malignancies and septic shock were associated with an increased risk of bacteremia.

## Background

Sepsis is a life-threatening organ dysfunction caused by a dysregulated host response to infection and involves a time-dependent pathological process requiring timely measures [1]. Among the community-acquired sepsis, pneumonia is a significant infection burden, with a short-term mortality rate of up to 50% in intensive care unit (ICU) patients [2–4]. To emphasize the emergent nature of sepsis management, the Surviving Sepsis Campaign published the Hour-1 bundle comprising lactate measurement, blood cultures, appropriate antimicrobials, resuscitation with volume, and use of vasopressors to initiate resuscitation within 1 h [5]. However, there are concerns that use of the Hour-1 bundle will lead to substantial overtreatment [6, 7].

In the recent American Thoracic Society (ATS)/Infectious Disease Society of America (IDSA) practice guideline for the management of CAP [8], blood cultures are only recommended in patients with severe CAP or those with risk factors for methicillin-resistant *Staphylococcus aureus* and *Pseudomonas aeruginosa*, with very low quality of evidence. Blood cultures are often performed for sepsis patients with severe CAP to identify the causative organisms and optimize antimicrobial therapy. However, the clinical benefit of this practice is uncertain and disputed, as there is a lack of high-quality studies supporting it [9–11]. Moreover, the impact of bacteremia on the outcomes of these patients is poorly understood.

In this study, we investigated the clinical characteristics and outcomes of severe CAP patients with bacteremia compared with subjects without bacteremia. In addition, we evaluated clinical factors that clinicians could use to target patients in whom blood cultures are most likely to yield a pathogen associated with bacteremia at the time of sepsis awareness.

## Methods

### Study design and population

This study was a secondary analysis of an ongoing nationwide multicenter prospective observational cohort study of sepsis patients between September 2019 and December 2020 [12]. Data were obtained from a sepsis registry created by the Korean Sepsis Alliance. Nineteen tertiary or university-affiliated hospitals in South Korea participated in the study. Patient registration and data collection protocols were previously presented [13, 14]. Briefly, the registry included patients 19 years of age and older diagnosed with sepsis based on the third International Consensus Definitions for Sepsis and Septic Shock (Sepsis-3) [1]. Patients were followed up until the time of hospital discharge or death.

We defined severe CAP as sepsis originating from a pulmonary infection. Our primary research objective was to evaluate the clinical effect of bacteremia in patients with severe CAP. Therefore, we included only the patients who underwent blood culture(s) according to the Surviving Sepsis Campaign bundle in the analysis [5]. We identified 5,718 patients with sepsis diagnosed in the ED during the study period. We excluded patients who did not have blood culture, or who had multiple or unclear sources of infection. In the analysis, we included only patients with sepsis due to a pulmonary infection and compared them between bacteremia and non-bacteremia groups [15].

The study was approved by the institutional review board of each participating hospital, including Samsung Medical Center (approval no. 2018–05-108), and the requirement for patient informed consent was waived due to the observational nature of the study. To protect individual privacy, the patient information was anonymized and de-identified before analysis.

### Data collection and definitions

Study coordinators in each participating center reviewed the electronic medical records of each patient and collected data using an electronic case report form (http://sepsis.crf.kr/). The following information was retrospectively collected: (1) demographic data of age, sex, body mass index, comorbidities, Charlson comorbidity index score, history of antibiotic administration or hospitalization for two or more of the 90 days before presenting to the ED, clinical frailty scale [16, 17], admission source (e.g., other hospitals, skilled nursing facility, or home), measure of illness severity using the Sequential Organ Failure Assessment (SOFA) score [18], physiological and laboratory measurements at the time of ED visit; (2) infection-related data of site of infection (e.g., lung, abdomen, urinary tract, or skin/soft tissue) and type of pathogen; (3) treatment data of implementation of nonsurgical control, use of adjunctive steroids, and admission to the ICU; (4) clinical outcomes of length of in-hospital stay and in-hospital death. Data regarding medical events and organ support for sepsis management during ICU stay were also collected.

Cultured pathogen was defined as any microorganism isolated from blood or sputum cultures [19, 20], obtained within two days of emergency department admission*.* Bacteremia was defined as the presence of bacteria in the blood culture. Among patients with severe CAP, blood cultures that grew coagulase-negative *staphylococci*, *Corynebacterium* spp., *Clostridium* spp., *Bacillus* spp., *Micrococcus* spp., and *Propionibacterium* spp. were defined as contaminated [9, 21]. Therefore, in patients with severe CAP, the bacteremia group comprised only patients with positive blood culture results excluding contaminants. Immunodeficiency defined as long-term use of steroids or other immunosuppressive drugs, except for hematologic malignancies or solid malignant tumor. Septic shock was defined as persistent arterial hypotension requiring a vasopressor to maintain mean arterial pressure ≥ 65 mmHg and a serum lactate level > 2 mmol/L despite adequate volume resuscitation [1]. The appropriateness of empirical treatment was determined according to the results of the drug susceptibility test or the guideline recommendations [22].

### Statistical analysis

Descriptive statistics were performed to compare the characteristics and clinical outcomes between the bacteremia and non-bacteremia groups. Continuous variables were presented as the median and interquartile range (IQR) and evaluated using the Mann–Whitney *U* test. Where applicable, categorical variables were expressed as number and percentages and analyzed using Chi-square or Fisher’s exact test. Logistic regression analysis was used to evaluate the risk factors for bacteremia. Variables shown to be clinically significant in previous studies [19] and obtained at the time of the ED admission were considered in the univariable and multivariable logistic regression models. The results were reported as the odds ratio (OR) of each variable with 95% confidence interval (CI). A two-sided *p*-value < 0.05 was considered statistically significant for all results. All analyses were performed using R Statistical Software (Version 3.2.5; R Foundation for Statistical Computing, Vienna, Austria).

## Results

**Fig. 1:**
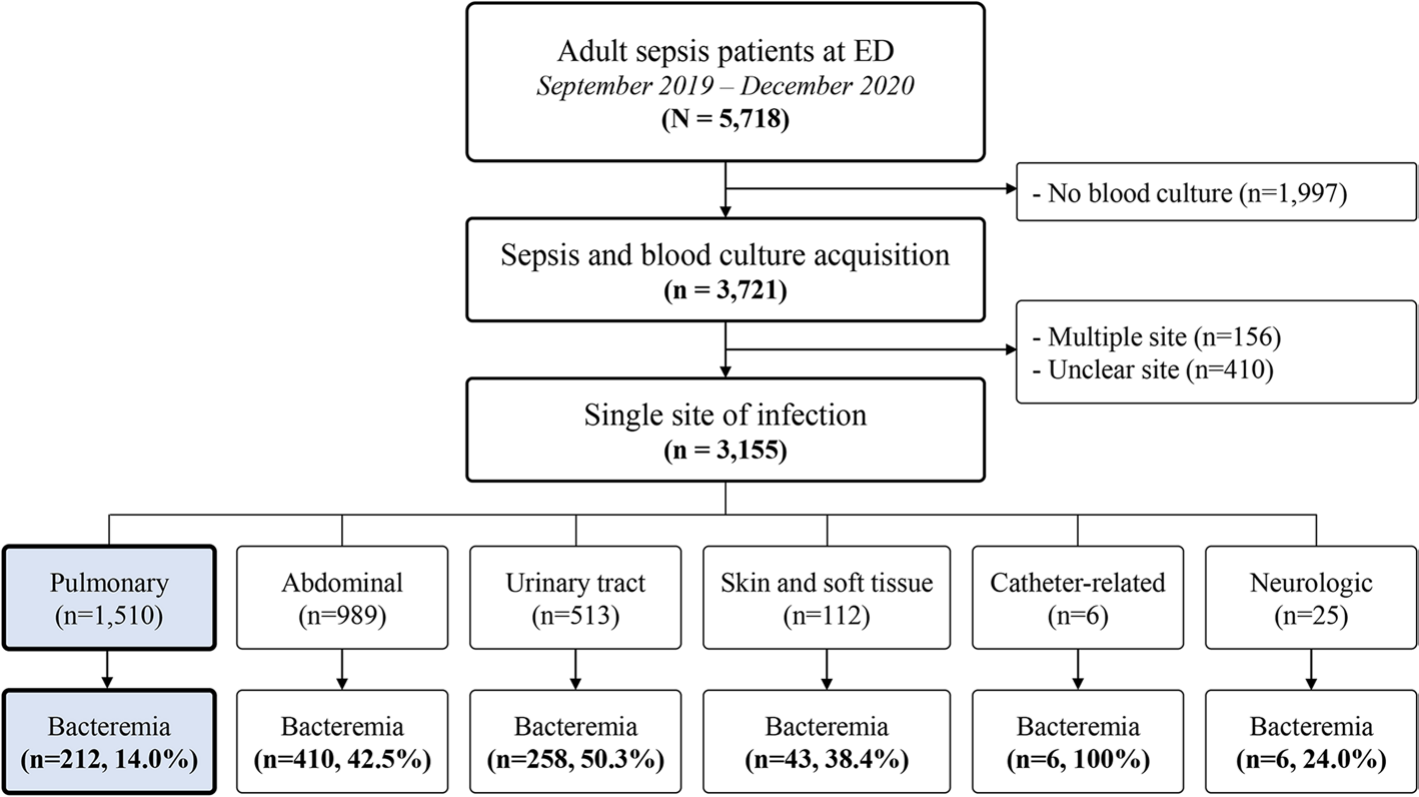
Scheme of sepsis patient distribution. ED = emergency department

During the study period, a total of 5,718 patients with community acquired sepsis were registered in the Korean Sepsis Alliance registry. Among them, 1,997 patients without blood cultures and 566 patients with suspected multiple sites or unclear site of infection were excluded. Of the 3,155 patients with community acquired sepsis and blood cultures, 1,510 (47.9%) sepsis patients caused by CAP were finally enrolled in this study. Out of 1,510 patients, 107 (7.1%) patients had blood cultures drawn after starting antibiotics. Pulmonary infection was most common suspected infection site, followed by abdominal and urinary tract infection (Fig. 1).

### Baseline patient characteristics

Among sepsis patients with CAP, bacteremia was identified in 212 (14.0%) patients. The clinical characteristics of the bacteremia and non-bacteremia groups are shown in Table 1. Age and body mass index were not significantly different between the two groups. Regarding comorbidities, hematologic malignancies were observed more frequently in the bacteremia group than in the non-bacteremia group, but less chronic lung disease was observed. Septic shock occurred more frequently in the bacteremia group than in the non-bacteremia group (27.4% vs. 14.8%; *p* < 0.001). The SOFA scores in the bacteremia and non-bacteremia groups were 7 (5–10) and 5 (3–7), respectively (*p* < 0.001).

**Table 1:** Comparison of baseline characteristics between patients with and without bacteremia

	**Total** **(*n* = 1,510)**	**Non-bacteremia** **(*n* = 1,298)**	**Bacteremia** **(*n* = 212)**	***p* value**
Age, years	74.0 (64.0–81.0)	74.0 (64.0–81.0)	73.0 (64.2–82.0)	0.683
Sex, male	1,039 (68.8)	897 (70.1)	142 (61.7)	0.013
Body mass index, kg/m^2^	21.0 (18.3–23.7)	20.9 (18.3–23.6)	21.3 (18.9–24.2)	0.180
Clinical frailty scale	5.0 (3.2–7.0)	5.0 (3.0–7.0)	6.0 (4.0–7.0)	0.026
History of antibiotics^a^	374 (24.7)	331 (25.5)	43 (20.3)	0.122
History of hospitalization^b^	468 (30.9)	406 (31.0)	62 (29.3)	0.576
Comorbidity				
Diabetes mellitus	509 (33.7)	431 (33.2)	78 (36.8)	0.309
Cardiovascular disease	404 (26.7)	346 (26.7)	58 (27.4)	0.867
Chronic lung disease	438 (29.0)	397 (30.6)	41 (19.3)	< 0.001
Chronic kidney disease^c^	178 (11.7)	144 (11.1)	34 (16.0)	0.051
Chronic neurological disease	318 (21.0)	269 (20.7)	49 (23.1)	0.415
Connective tissue disease	39 (2.5)	32 (2.5)	7 (3.3%)	0.482
Solid malignant tumor	500 (33.1)	442 (34.1)	58 (27.4)	0.066
Hematologic malignancy	86 (5.7)	62 (4.8)	24 (11.3)	< 0.001
Immunodeficiency	44 (2.9)	35 (2.7)	9 (4.3)	0.266
Charlson comorbidity index	5.0 (4.0–7.0)	5.0 (4.0–7.0)	5.0 (4.0–7.0)	0.812
Septic shock	250 (16.5)	192 (14.8)	58 (27.4)	< 0.001
SOFA score	5.0 (3.0–7.0)	5.0 (3.0–7.0)	7.0 (5.0–10.0)	< 0.001
Vital signs				
Mean blood pressure, mmHg	73.3 (63.3–93.3)	73.7 (64.0–94.0)	66.7 (60.0–81.0)	< 0.001
Heart rate, /min	110.0 (94.0–126.0)	110.0 (94.0–125.0)	113.0 (98.0–130.0)	0.048
Respiratory rate, /min	24.0 (22.0–28.0)	24.0 (22.0–28.0)	24.0 (22.0–30.0)	0.221
Laboratory findings				
White blood cell count,10^3^/μL	11.0 (7.0–15.4)	11.1 (7.2–15.6)	10.4 (5.1–14.1)	0.008
Hemoglobin, g/dL	11.3 (9.6–13.1)	11.4 (9.7–13.2)	10.6 (8.8–12.7)	< 0.001
Platelets, × 10^3^/μL	194 (124–273)	204.0 (135.0–278.7)	145.0 (77.5–241.5)	< 0.001
ANC, × 10^3^/μL	8.8 (5.3–12.9)	8.9 (5.5–13.2)	8.7 (3.5– 11.6)	0.023
Creatinine, mg/dL	1.2 (0.8–1.9)	1.2 (0.8–1.8)	1.5 (1.0–2.5)	< 0.001
Total bilirubin, mg/dL	0.7 (0.5–1.2)	0.7 (0.5–1.1)	1 (0.6–1.6)	< 0.001
Prothrombin time, INR	1.1 (1.0– 1.3)	1.1 (1.0–1.3)	1.2 (1.1–1.4)	< 0.001
Albumin, g/dL	3.1 (2.6–3.6)	3.2 (2.7–3.6)	2.8 (2.4–3.2)	< 0.001
C-reactive protein, mg/dL	10.6 (4.5 –19.4)	9.9 (4.2–18.4)	15.7 (8.3–25.4)	< 0.001
Procalcitonin, mmol/L	1.0 (0.2–6.4)	0.8 (0.2–4.6)	4.2 (1.0–24.1)	< 0.001
Lactate, mmol/L	2.4 (1.5–4.5)	2.3 (1.4–4.0)	3.8 (2.0–6.2)	< 0.001

Values are median (interquartile range, IQR) or number (%)

*ANC* absolute neutrophil count, *SOFA* Sequential Organ Failure Assessment

^a^History of antibiotic prescription within the past 30 days

^b^Hospitalization for ≥ 2 of the past 90 days

^c^Chronic kidney disease is defined as either kidney damage or GFR < 60 mL/min/1.73 m^2^ for 3 months

### Clinical factors associated with bacteremia

To determine clinical factors associated with bacteremia that were present at the time of suspected sepsis in the emergency department, univariable logistic regression analysis was performed using 11 clinical characteristics that were obtained at the time of emergency department admission (Table 2). Logistic regression analysis showed that underlying hematologic malignancy and septic shock were associated with bacteremia in severe CAP. However, chronic lung disease was associated with a decreased risk of bacteremia (OR 0.54; adjusted OR 0.55; 95% CI 0.37–0.78; *p* = 0.001).

**Table 2:** Logistic regression analysis of clinical characteristics at the time sepsis was suspected in the emergency department for bacteremia prediction

	**Univariable model**		**Multivariable model**	
	**OR (95% CI)**	***p* value**	**OR (95% CI)**	***p* value**
Age, > 70 years	0.99 (0.74–1.33)	0.936	1.01 (0.74–1.39)	0.948
Sex, female	1.47 (1.09–1.99)	0.011	1.34 (0.98–1.82)	0.068
Clinical frailty scale score > 4	1.29 (0.96–1.74)	0.100	1.34 (0.97–1.85)	0.075
Diabetes mellitus	1.13 (0.84–1.52)	0.306	1.09 (0.79–1.49)	0.590
Chronic lung disease	0.54 (0.38–0.77)	< 0.001	0.55 (0.37–0.78)	0.001
Chronic liver disease	1.42 (0.83–2.44)	0.196	1.43 (0.78–2.47)	0.224
Chronic kidney disease	1.53 (1.01–2.27)	0.040	1.49 (0.96–2.26)	0.068
Solid malignant tumor	0.73 (0.52–1.00)	0.056	0.82 (0.58–1.15)	0.256
Hematologic malignancy	2.54 (1.52–4.13)	< 0.001	2.44 (1.43–4.05)	< 0.001
Immunodeficiency	1.60 (0.71–3.24)	0.218	1.23 (0.52–2.64)	0.619
Septic shock	2.17 (1.54–3.03)	< 0.001	2.22 (1.56–3.12)	< 0.001

*CI* confidence interval, *OR* odds ratio

### Treatment characteristics and clinical outcomes

The appropriateness of initial empiric antibiotics in both groups was 91.8% and 87.7%, respectively (*p* = 0.050). Overall, 36 (2.4%) patients received pleural percutaneous catheter drainage insertion, and 323 (21.3%) received adjunctive steroid therapy (Table 3). The statistically significant difference was not observed in ICU admission (43.9% vs. 38.7%; *p* = 0.174) or hospital length of stay (12.5 days vs. 12.0 days; *p* = 0.976). Of the 595 patients admitted to the ICU, 400 (67.2%) patients received mechanical ventilator support and 15 (2.5%) patients underwent extracorporeal membrane oxygenation support. However, hospital mortality was significantly higher in the bacteremia group than in the non-bacteremia group (40.6% vs. 27.3%; *p* < 0.001).

**Table 3:** Comparison of management and clinical outcomes between patients with and without bacteremia

	**Overall** **(*n* = 1,510)**	**Non-bacteremia** **(*n* = 1,298)**	**Bacteremia** **(*n* = 212)**	***p* value**
Appropriateness of initial antibiotics				0.050
Appropriate	1,377 (91.2)	1,191 (91.8)	186 (87.7)	
Inappropriate	118 (7.8)	93 (7.2)	25 (11.8)	
Not available	15 (1.0)	14 (1.1)	1 (0.4)	
Pleural PCD insertion	36 (2.4)	33 (2.6)	3 (1.3)	0.396
Adjunctive steroid treatment	323 (21.3)	268 (20.6)	55 (25.9)	0.098
ICU admission	595 (39.4)	502 (38.7)	93 (43.9)	0.174
ICU management				
Mechanical ventilation	400 (67.2)	332 (66.0)	68 (73.1)	0.222
ECMO	15 (2.5)	15 (3.0)	0 (0)	0.151
Renal replacement therapy	128 (21.4)	101(20.1)	27 (29.0)	0.073
Hemoperfusion	17 (2.8)	16 (3.2)	1 (1.0)	0.357
Clinical outcomes				
Hospital length of stay	12.0 (6.0–20.0)	12.0 (6.0–20.0)	12.5 (5.0–23.0)	0.976
Hospital mortality	441 (29.2)	355 (27.3)	86 (40.6)	< 0.001

Values are median (interquartile range, IQR) or number (%)

*ECMO* extracorporeal membrane oxygenation, *ICU* intensive care unit, *PCD* percutaneous catheter drainage

### Bacterial pathogens in sepsis patients with CAP

The bacterial pathogens in sepsis patients with CAP isolated from blood and sputum culture are shown in Tables 4 and 5, respectively. The most prevalent pathogen in blood culture was *Klebsiella pneumoniae* followed by *Escherichia coli* in gram-negative pathogens (Table 4). Additionally, among gram-positive pathogens was *Staphylococcus aureus* followed by *Streptococcus pneumoniae.* In the pathogens isolated from the respiratory tract, there was no statistical difference between the two groups except for *Coagulase-negative staphylococci* and *Escherichia coli* (Table 5).

**Table 4:** Isolation of bacterial pathogens from blood culture in bacteremia patients

	***N*** **(%)**
Pathogens	*n* = 212
*Klebsiella pneumoniae*	66 (28.7)
*Escherichia coli*	38 (16.5)
*Staphylococcus aureus*	33 (14.4)
*Pseudomonas aeruginosa*	17 (7.4)
*Streptococcus pneumoniae*	13 (5.7)
*Acinetobacter baumannii*	9 (7.0)
*Enterococcus faecium*	4 (1.7)
*Enterococcus faecalis*	3 (1.3)
*Listeria monocytogenes*	3 (1.3)
*Others*^*a*^	26 (12.3)

Values are presented as number (%)

^a^Others include *Bacteroides ovatus* (*n* = 1)*, Citrobacter spp.* (*n* = 2)*, Cutibacterium acnes* (*n* = 1)*, Enterobacter cloacae complex* (*n* = 1)*, Enterococcus durans* (*n* = 1)*, Fusobacterium nucleatum subsp. nucleatum* (*n* = 1)*, Moraxella catarrhalis* (*n* = 1)*, Morganella morganii* (*n* = 1)*, Microbacterium oxydans* (*n* = 1)*, Pantoea dispersa* (*n* = 1)*, Parvimonas micra* (*n* = 1)*, Proteus spp.* (*n* = 2)*, Providencia rettgeri* (*n* = 1)*, Serratia marcescens* (*n* = 1)*, Streptococcus constellatus* (*n* = 2)*, Streptococcus dysgalactiae* (*n* = 1)*, Streptococcus intermedius* (*n* = 2)*, Streptococcus lutetiensis* (*n* = 1)*, Streptococcus parasanguis* (*n* = 1)*, Streptococcus salivarius* (*n* = 1)*, Viridans group streptococci* (*n* = 1)*,* and *Weissella confusa* (*n* = 1)

**Table 5:** Isolation of bacterial pathogens from the respiratory tract

	**Non-bacteremia** **(*n* = 261)**	**Bacteremia** **(*n* = 44)**	***p* value**
Gram-positive pathogen			
*Staphylococcus aureus*	52 (19.9)	11 (25.0)	0.570
*Streptococcus pneumoniae*	27 (10.3)	3 (6.8)	0.593
*Corynebacterium striatum*	11 (4.2)	2 (4.6)	> 0.99
*Coagulase-negative staphylococci*	0 (0)	6 (13.6)	< 0.001
* Enterococcus faecalis*	3 (1.2)	2 (4.6)	0.318
* Enterococcus faecium*	4 (1.5)	1 (2.3)	> 0.99
Gram-negative pathogen			
*Klebsiella pneumoniae*	80 (30.7)	18 (40.9)	0.241
*Pseudomonas aeruginosa*	37 (14.2)	9 (20.5)	0.396
*Acinetobacter baumannii*	22 (8.4)	6 (13.6)	0.410
*Escherichia coli*	17 (6.5)	8 (18.2)	0.021
*Haemophilus influenza*	15 (5.8)	0 (0)	0.210
*Proteus spp.*	4 (1.5)	2 (4.6)	0.457

Values are presented as numbers (%)

## Discussion

In the present study, we investigated the clinical characteristics and effects of bacteremia on severe CAP. We found that severe CAP patients with bacteremia had increased mortality compared with subjects without bacteremia. The bacteremia incidence was 14.0%, and hematologic malignancies and septic shock were associated with bacteremia occurrence.

Previously, the bacteremia incidence was suggested to vary based on infection site. In previous bacteremia studies, intra-abdominal infection was the most common cause of bacteremia, followed by urinary tract infection with increased bacteremia up to 24–26% [23, 24]. In the present study, the incidence of bacteremia was 42.5% in intra-abdominal infection and 50.3% in urinary tract infection in sepsis patients.

Positive blood cultures were found in approximately 5%–14% of CAP patients [25, 26], similar to the 14.0% identified in this study. Due to low bacteremia incidence in CAP, many clinicians question the need to perform blood cultures for all pneumonia patients. For select CAP patients requiring blood culture, some studies evaluated risk factors for bacteremia [9, 27]. Metersky et al. found that prior antibiotics, underlying liver disease, decreased systolic blood pressure, decreased or increased body temperature, increased pulse rate, increased blood urea nitrogen, decreased serum sodium, and leukocytosis or leukopenia were associated with bacteremia in patients with CAP [9]. Lee et al. reported that low blood pressure, increased pulse rate, decreased or increased body temperature, leukocytosis or leukopenia, thrombocytopenia, decreased serum albumin, and increased serum C-reactive protein were associated with bacteremia in patients with CAP at the EDs [27]. Previous studies included laboratory test results that could be delayed by hours before results were available, making these clinical factors impractical in the real world, especially sepsis patients.

One of the components of the Hour-1 bundle is obtaining blood culture. Although blood culture is considered the most sensitive method for detection of bacteremia in critically ill patients, routine blood cultures in a crowded ED is not an efficient use of resources [28, 29].

A recent multicenter observational study of sepsis in the ED also reported that only 58% of sepsis patients obtained blood cultures within 1 h after ED visit [30]. In this study, we found several clinical factors that can be easily assessed at recognition of sepsis including several co-morbidities and shock status. We also found that underlying clinical condition are closely associated with increased bacteremia risk in severe CAP. Therefore, blood cultures should be performed in patients at risk for bacteremia, concomitant with sputum culture in patients with severe CAP. Previous studies did not find the impact of comorbidities on the risk of bacteremia [12, 19]. However, in this study, we identified hematologic malignancy as important risk factors for bacteremia. A potential explanation for the lack of significant differences in comorbidities in previous studies may be related to differences in the study population and the comparison groups. Jeon K et al. [12] compared sepsis and septic shock patients regardless of infection site. Margret M et al. [19]compared patients according to the presence of bacteremia among those with nosocomial pneumonia. However, in our study, we compared patients according to the presence of bacteremia among those with severe CAP. Some comorbidities may predispose patients to specific types of pathogens that are more likely to cause bacteremia. For instance, diabetes mellitus has been shown to increase the risk of *Staphylococcus aureus* bacteremia [31, 32]. Therefore, further studies are needed to elucidate the role of comorbidities in the pathogenesis and outcome of bacteremia in patients with severe CAP.

The most common pathogen in CAP is *Streptococcus pneumoniae*, which accounts for two-thirds of bacterial pneumonia [33], followed by *Mycoplasma pneumoniae* and *Haemophilus influenzae.* Lanks et al. demonstrated a relationship between bacterial pathogens and CAP severity. In their study, *S. aureus, Legionella*, and *H. influenzae* were associated with increased disease severity [34]. However, our study showed different results, with *K. pneumoniae* being the most common pathogen regardless of bacteremia, followed by *E. coli* and *S. aureus* in the bacteremia group and *S. aureus* and *P. aeruginosa* in the non-bacteremia group. This is likely due to differences in geographic and hospital-specific characteristics [35], and reaffirms that empiric antibiotic administration in consideration of geographic differences.

The study was conducted during the coronavirus disease (COVID)-19 pandemic, which may have changed the epidemiology of bacterial pneumonia due to infection control practices. This could introduce a selection bias in our results. A recent study of COVID-19 pneumonia patients by Rouze A, et al. reported that most of the early identification of bacterial coinfection was related to gram-positive cocci, predominantly *S. aureus*, and was associated with increased for 28-day mortality [36]. They suggested that the lack of estimates for the prevalence of bacterial infections and the inability to exclude bacterial involvement definitively have led to the widespread prescription of empirical antimicrobial therapy. Moreover, another study also found a high rate of early bacterial infection during severe COVID-19 pneumonia, and a high rate of *S. aureus* [37]. In our study, *S. aureus* also showed the highest frequency among gram-positive cocci, indicating that community-acquired infections are influenced not only by geographical factors but also by environmental factors such as prevalence of bacterial pathogen.

The definition and exclusion of bacterial contamination in bacteremia cases with non-pulmonary sources are inconsistent. As previously stated, coagulase-negative staphylococci, Corynebacterium spp., Clostridium spp., Bacillus spp., Micrococcus spp., and Propionibacterium spp. are considered contaminants in blood cultures of CAP patients. In contrast, they are often the actual pathogens in non-pulmonary sources. This discrepancy may result in a higher rate of bacteremia for non-pulmonary infections than for pulmonary infections. Thus, our data are not readily comparable with the reported incidence of bacteremia in non-pulmonary infections.

Bacteremia is a significant cause of morbidity and mortality in critically ill patients [38]. Although only a few studies exist on bacteremia and CAP, our study, like previous studies on nosocomial blood stream infections, found that in-hospital mortality was significantly higher in the bacteremia group than in the non-bacteremia group. This finding should be validated in future studies of CAP, depending on disease severity.

Potential limitations should be acknowledged to fully appreciate the results of our study. First, as this study was conducted only in patients from 19 hospitals in South Korea, the results might not be generalizable to different regions. Second, this study included only patients who were diagnosed with sepsis at presentation to the ED. Thus, the results may not be generalizable to patients with sepsis in the hospital. Third, this study included only severe CAP. Therefore, we could not be represent all CAP patients’ clinical characteristics and outcomes. Fourth, we did not exclude any bacterial species isolated from sputum, which might introduce the possibility of chronic carriage. The isolation of some pathogens from blood cultures suggests the possibility of alternative sources of infection besides pneumonia. Fifth, the study coincided with the COVID-19 pandemic, which may have influenced the etiology of pneumonia due to infection control measures that reduced the exposure to community pathogens such as *S. pneumoniae*. This factor should be taken into account when interpreting the results. Sixth, we did not include information on the chest images of severe CAP patients. Further studies including detailed knowledge of pneumonia would be informative. Finally, we observed that 107 (7.1%) patients obtained blood cultures after starting antibiotics, which could affect the identification of causative organisms that would be isolated. In addition, we did not investigate the frequency of functional or anatomical asplenia, which is a significant risk factor for bacteremia caused by encapsulated bacteria.

## Conclusion

The incidence of bacteremia in severe CAP was low at 14.0%, but the occurrence of bacteremia was associated with increased hospital mortality. Bacteremia in severe CAP was associated with hematologic malignancy and septic shock. These easily assessable clinical factors have important implications for the initial assessment and management severe CAP patients at ED.

## Data Availability

The data supporting this study’s findings are available on request from the corresponding author. The data are not publicly available due to privacy or ethical restrictions.

## Abbreviations

CAP: Community-acquired pneumonia
COVID: Coronavirus disease
CI: Confidence interval
ED: Emergency department
IQR: Interquartile range
OR: Odds ratio
SOFA: Sequential Organ Failure Assessment
